# Vascularized bone graft from the second metacarpal base for trapeziometacarpal joint arthrodesis

**DOI:** 10.1080/23320885.2018.1493930

**Published:** 2018-08-07

**Authors:** Akito Nakanishi, Kenji Kawamura, Shohei Omokawa, Takamasa Shimizu, Yasuhito Tanaka

**Affiliations:** a Nara Medical University, Department of Orthopedic Surgery, Kashihara, Nara, Japan;; b Nara Medical University, Department of Hand Surgery, Kashihara, Nara, Japan

**Keywords:** Vascularized bone graft, the second metacarpal base, trapeziometacarpal joint, non-union, osteoarthritis

## Abstract

Three female patients underwent TMC joint arthrodesis using a vascularized bone graft from the second metacarpal base. Surgical indications were nonunion after failed TMC joint arthrodesis and osteoarthritis and severe osteoporosis. All cases achieved early bone union, and marked postoperative improvement in the VAS and DASH scores.

## Introduction

Hori et al. reported successful blood vessel transfer to necrotic bone using a dorsal metacarpal artery and veins in Kienböck disease and Preiser disease [1]. Furthermore, satisfactory clinical results have been reported using a vascularized second metacarpal base bone graft for the treatment of scaphoid nonunion and Kienböck disease [2]. Sawaizumi et al. modified this procedure, and performed this graft from the palmar side for scaphoid nonunion accompanied by dorsal intercalated segment instability [3–4]. However, there are no previous reports on the application of this procedure in patients with trapeziometacarpal (TMC) osteoarthritis. We used this procedure in patients with nonunion after failed arthrodesis of the TMC joint or with TMC osteoarthritis and severe osteoporosis. The purpose of the present article was to introduce the procedure of TMC joint arthrodesis using a vascularized bone graft based on the second metacarpal artery and veins, and to assess the clinical and radiological outcomes in three cases.

## Case Report

From February 2011 to January 2014, three patients (three females; average age, 71 ± 11 years) underwent TMC joint arthrodesis using a vascularized bone graft from the second metacarpal base. The indications for TMC joint arthrodesis were painful nonunion after arthrodesis of the TMC joint (n = 1), and TMC joint arthrosis with severe osteoporosis (n = 2). These three cases were evaluated at a minimum of 3 years postoperatively. Bone union was defined as the appearance of a trabecular connection showing bone healing at the TMC joint [5]. Bone union was obtained in all cases. The average bone healing time was 6.7 weeks (Table 1). The VAS and DASH scores were markedly improved in all cases (Table 1). There were no complications at the donor site at final follow-up in all three cases.

**Table 1.** Data for patients.

Patient	Age	Sex	Diagnosis	Bone union period	VAS		DASH	
					Pre	Post	Pre	Post
1	55	Female	Non-union	6w	80	10	75	5
2	78	Female	TMC osteo-arthritis	6w	85	5	50	9.5
3	80	Female	TMC osteo-arthritis	8w	75	10	32.5	9.2

All participants provided written informed consent, and the present study was approved by the Institutional Review Board of our institution.

### Operative technique

**Figure 1.**
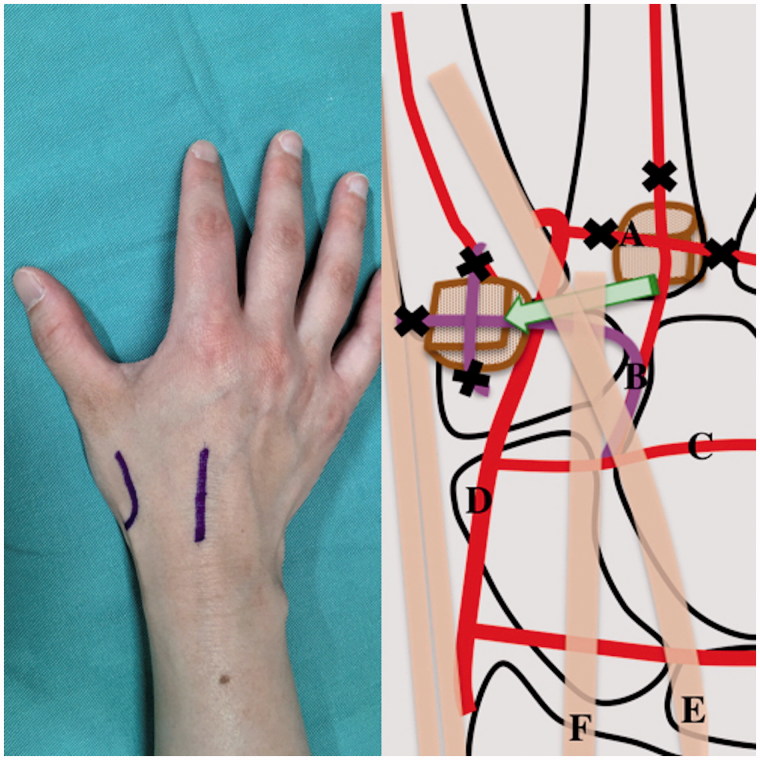
Approach method and diagram of surgical procedure. A, basal metacarpal arch; B, second dorsal metacarpal artery; C, dorsal intercarpal arch; D, radial artery; E, extensor pollicis longus; F, extensor carpi radialis.

All procedures were performed with the patient under general anesthesia, and a pneumatic tourniquet was used in each case. On the dorsal side, a 3 cm curved skin incision was made over the second TMC joint (Figure 1). The surgical approach was a dorsal approach through the APL/EPB interval. After retracting the superficial radial nerve branches and exposing the TMC joint, the fibrous tissue and screws at the nonunion site were removed in the patient with failed arthrodesis, and the damaged articular cartilage and subchondral bone in the other two patients were sufficiently excised to expose the underlying cancellous bone. Another 3 cm skin incision was made over the second metacarpal base (Figure 1). The second dorsal metacarpal artery and concomitant veins branching from the dorsal intercarpal artery were identified and dissected at the shaft of the second metacarpal bone, while the basal metacarpal arch was coagulated. The vascular pedicle was then elevated proximally with the periosteum and bone cortex and cancellous bone at the base of the second metacarpal bone (pedicle length 35 mm, vascularized graft segment 12 × 10 × 10 mm). The pedicle bone graft was transferred and placed in the first carpometacarpal joint underneath the extensor pollicis longus tendon and the extensor carpi radialis tendon (Figure 1). Bleeding from the elevated cancellous bone was observed after releasing the pneumatic tourniquet, indicating good vascularization. Finally, the pedicle graft and the TMC joint were fixed with a T-shaped plate or two cannulated headless compression screws. There was no additional procedure performed at the donor site after harvesting the bone flap. Postoperatively, a short arm cast was used for immobilization for 4 weeks. After cast removal, thumb and hand therapy was started, aiming at regaining wrist and thumb mobilization.

**Figure 2.**
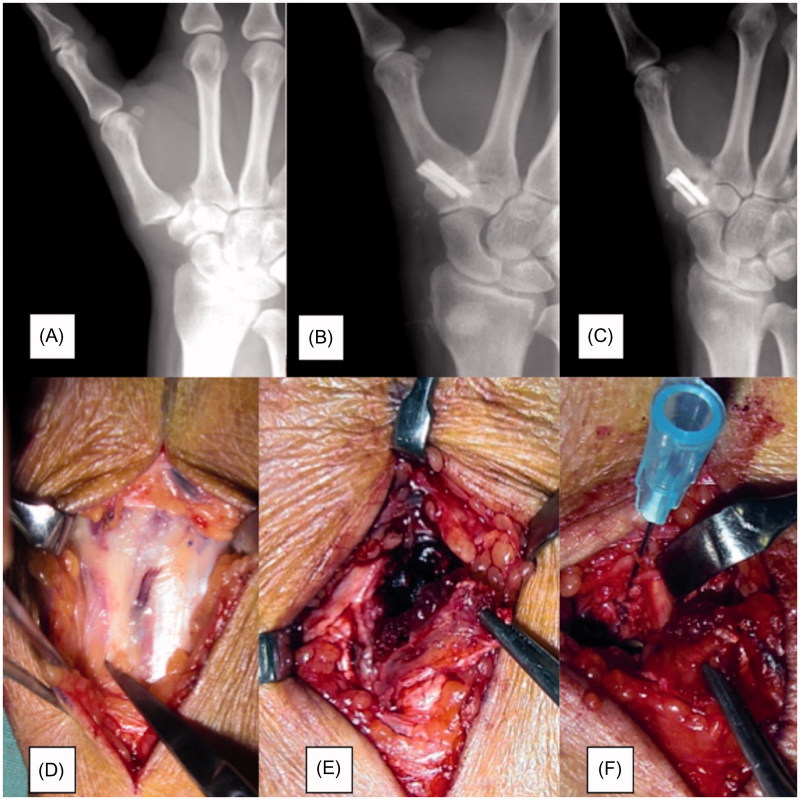
Case 1: a 58-year-old female. (A) Preoperative radiograph (dorsoplantar view) of the right hand showing dislocation of the first carpometacarpal joint. (B) Immediately postoperative radiograph after the first surgery. (C) Radiography at 6 months after the first surgery showing nonunion. (D) Intraoperative photograph of the second metacarpal artery. (E) Photograph of the vascularized bone graft at the time of harvesting. (F) Photograph of the vascularized bone graft at the time of transferal to the first carpometacarpal joint.

**Figure 3.**
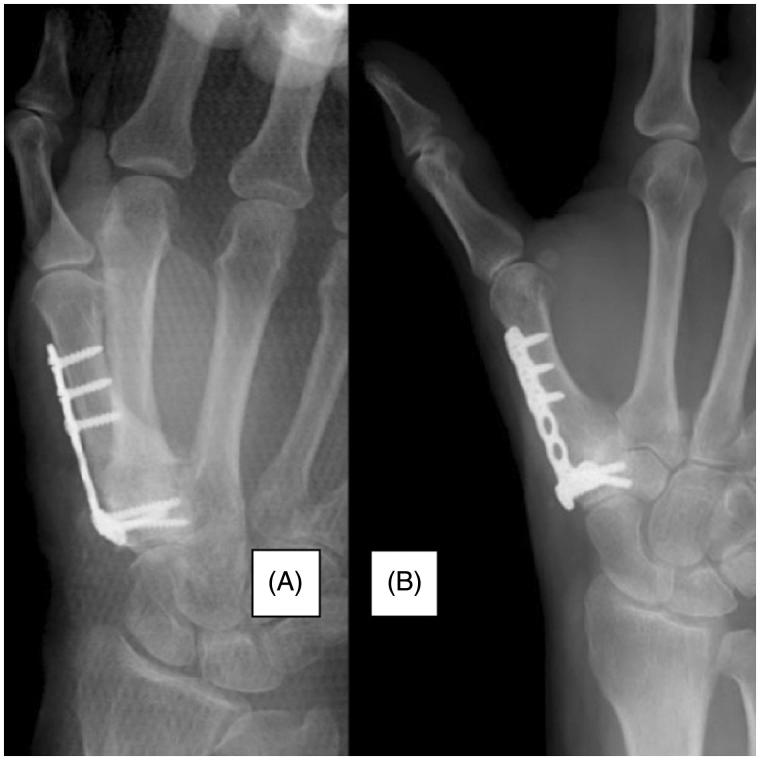
Case 1: a 58-year-old female. (A) Immediately postoperative radiograph of the right hand (dorsoplantar view) after the second surgery. (B) At 2 months after the second surgery, radiography confirmed bony union.

Case 1: A 58-year-old right-hand dominant female underwent the first carpometacarpal arthrodesis using two cannulated compression headless screws. Postoperatively, strong pinch movement was prohibited for 4 weeks, and range of motion exercises were undertaken. There was persistent pain in the fixed joint from 6 weeks postoperatively; hence, a thumb spica cast was applied for immobilization. However, the pain was still present at 6 months postoperatively, and radiography showed nonunion and loosening around the screws. The patient was treated with a vascularized bone graft from the second metacarpal base and plate fixation (Figures 2 and 3). The length of surgery was about 90 minutes. Radiographs showed bone union at 6 weeks postoperatively, and the Kapandji score was 7. Tip pinch and side pinch were restored to 92% of the healthy side, and the VAS and DASH scores were improved (Table 1). The patient returned to unrestricted daily activity at 3 months after the second surgery.

**Figure 4.**
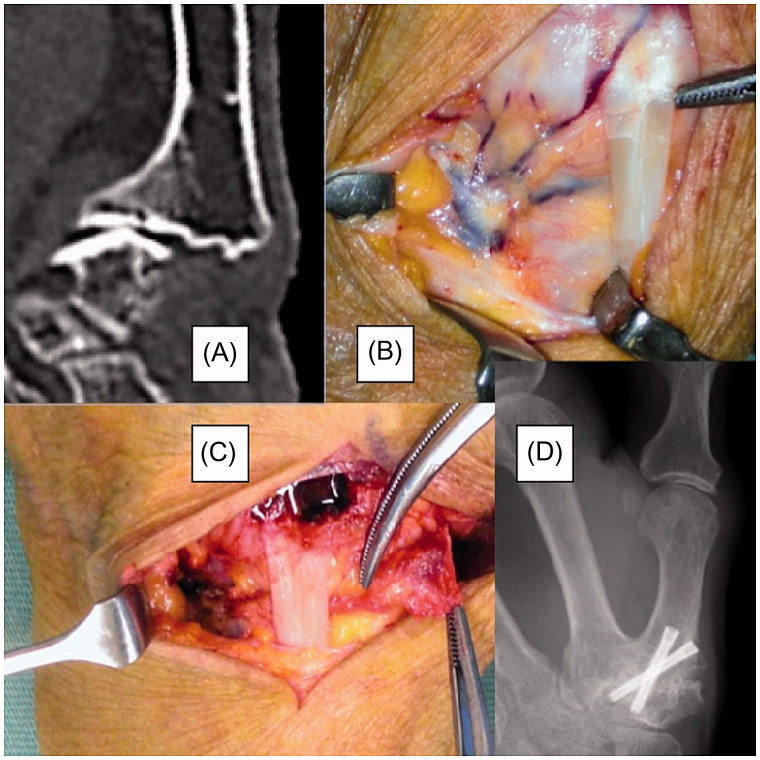
Case 2: a 78-year-old female. (A) Preoperative computed tomography showing thumb carpometacarpal arthritis with marked osteoporosis. (B) Intraoperative photograph of the second metacarpal artery. (C) Photograph of the vascularized bone graft at the time of transferal to the first carpometacarpal joint. (D) Radiography at 2 months postoperatively confirmed bony union.

Case 2: A 78-year-old right-hand dominant female had a 7-year history of left thumb pain at night. Physical examination and radiography revealed osteoarthritis of the TMC joint. Radiographs showed degenerative arthritis (Eaton classification stage III). Following failure of conservative treatment, the patient underwent arthrodesis using a vascularized second metacarpal base bone graft and two crossed cannulated compression screws (Figure 4). The length of surgery was about 60 minutes. Radiography showed successful bone union at 6 weeks postoperatively, and the Kapandji score was 7. Tip pinch and side pinch were restored to 92% of the healthy side, and the VAS and DASH scores were improved (Table 1). At 6 weeks postoperatively, the patient could perform all activities of daily living without restriction.

## Discussion

The first vascularized pisiform bone graft was reported in 1971 [6]. Hori et al. then successfully performed vascular bundle implantation to necrotic bone [1]. Following these publications, transfer of a live bone graft with its nutrient vascular pedicle was performed with satisfactory clinical outcomes in patients with recalcitrant nonunion or osteonecrosis. Makino reported a case in which a vascularized second metacarpal base bone graft was successfully used to treat scaphoid nonunion and Kienböck’s disease [2]. We used this procedure to obtain early and reliable bone union in arthrodesis of the TMC joint in three cases.

Carroll advised against TMC joint arthrodesis in elderly patients due to the risk of progression of pantrapezial arthritis [7]. However, according to recent papers of Rizzo M [5] and Hattori Y [8], the indication of TMC joint arthrodesis has been expanded to include older. Previous studies have reported nonunion rates of 7% to 47% after TMC joint arthrodesis [9–11]. Even in the initial treatment of TMC joint osteoarthritis, we consider that a vascularized bone graft may provide a good surgical option for joint fusion surgery in elderly patients with severe osteoporosis or in those who are smokers, as there have been no donor site complications reported after this procedure, and the donor and recipient sites are closely located. In contrast, it takes a long time to obtain bone union after carpometacarpal thumb arthrodesis with a conventional bone graft in elderly patients [8].

Previous anatomical and clinical studies report that the second metacarpal artery and veins are consistent, and thus these vessels can be easily harvested with a periosteal flap [2–4]. The size of the harvested bone is sufficient to achieve bone union at the TMC joint, and the adjacent location of the donor site may enable relatively less invasive surgery than that using other vascularized bone graft donor sites. In conclusion, a vascularized second metacarpal base bone graft is a good option in cases of nonunion after arthrodesis of the TMC joint, and for arthrodesis in those with severe osteoporosis or in those who are smokers.
